# A case of hyperventilation leading to apnea and desaturation in PACU

**DOI:** 10.1186/s12871-019-0827-2

**Published:** 2019-08-14

**Authors:** Parineeta Thapa, Asish Subedi, Anjali Poudel, Pankaj Baral

**Affiliations:** Department of Anaesthesiology and Critical Care, B. P. Koirala Institute of Health Sciences, Dharan, Koshi Nepal

**Keywords:** Apnea, Hyperventilation, Hypoxia, Recovery room

## Abstract

**Background:**

Respiratory adverse events are not uncommon in the post-anesthesia care unit (PACU) following general anesthesia. In this regard, hyperventilation leading to apnea and desaturation is a rare entity. Here we have reported a case of a 15-year-old girl who, following an uneventful general anesthesia, developed severe hyperventilation leading to apnea and desaturation in the PACU.

**Case presentation:**

The 15-year-old girl underwent cortical mastoidectomy under general anesthesia. After a smooth anesthesia and an uneventful early recovery, she developed hyperventilation after about 15 min in the PACU. The symptom was severe enough to lead to apnea, desaturation and severe respiratory alkalosis. She required bag and mask ventilation and the symptoms resolved only transiently with propofol sedation. Finally, she responded to intravenous haloperidol and did not have any further episode after receiving haloperidol.

**Conclusion:**

Hyperventilation after a smooth recovery from anesthesia is not a common presentation. In this article we have tried to discuss the possible cause of such symptom in our patient and how we successfully managed this case. We have also proposed an algorithmic approach to diagnose and manage such cases in the PACU.

## Background

Respiratory complications are not uncommon in the immediate post-operative period. We frequently come across problems like airway obstruction, desaturation, hypoventilation, ineffective ventilation due to inadequate reversal of neuromuscular blocking agents, etc. But hyperventilation as a sole presentation following smooth early recovery from general anesthesia is not a frequent encounter. We recently faced such a case where the patient, after an uneventful general anesthesia and early recovery, developed severe hyperventilation leading to apnea and desaturation. We ruled out and managed possible organic causes and even considered psychological cause for such symptom. Here we present this case as well as discuss the possible causes and an approach to such cases if it occurs in the post-anesthesia care unit (PACU).

## Case presentation

A 15-year-old girl was posted for cortical mastoidectomy with tympanoplasty for chronic suppurative otitis media under general anesthesia. She was thin built with body weight of 40 kg and body mass index 18 kg/m^2^, American Society of Anesthesiologists Physical Status (ASA-PS) I, with no significant family or personal medical history. Preoperative investigations were unremarkable. She was anxious about the surgery, for which she was counselled and advised oral diazepam 5 mg on the night before and 2 h prior to surgery.

The patient was still anxious when she arrived in the operation theatre, so she was again counselled and reassured. Anesthesia was induced with intravenous (IV) fentanyl 80 μg, propofol 80 mg and vecuronium 4 mg. After tracheal intubation, anesthesia was maintained with isoflurane in 50% oxygen in air, with supplemental doses of vecuronium to maintain muscle relaxation. Paracetamol 750 mg IV infusion and IV dexamethasone 4 mg was given after induction of anesthesia. During the 2 h of surgical period her vital signs were stable. Ventilation was adjusted to keep end tidal CO_2_ (ETCO_2_) between 35 and 40 mmHg. She received 600 ml of Ringers lactate intraoperatively, and the blood loss was insignificant. IV ondansetron 4 mg was given at the end of the surgery. After the completion of surgery, residual neuromuscular blockade was reversed with IV neostigmine 2 mg and glycopyrrolate 0.4 mg. Once she regained consciousness, obeyed commands, and had regular spontaneous respiration with adequate tidal volume, her trachea was extubated and she was shifted to the PACU. Supplemental oxygen at 5 L/min was provided via facemask.

After 15 min in the PACU, the patient complained of difficulty in breathing and became agitated. She started hyperventilating with deep breaths at the respiratory rate (RR) of 60–70 breaths/min. Her heart rate (HR) and blood pressure (BP) started to increase and reached a maximum of 180 beats/min and 180/70 mmHg respectively. Initially, we suspected it to be agitation due to pain and gave her fentanyl 20 μg IV bolus, but there was no improvement. We tried to support the ventilation using Bain’s circuit and anatomical face mask with oxygen flow at 10 L/min. Suspecting recurarization, 0.5 mg neostigmine and 0.1 mg glycopyrrolate IV was given. But her respiratory rate did not settle. So, we planned to sedate her with IV propofol 20 mg. She became calm with normal breathing pattern and her HR settled to 100–110 beats/min. But once the patient was awake from the effect of propofol, the symptoms reoccurred. Suspecting full bladder, bladder catheterization was done and urine was drained. As the symptom did not improve, IV propofol 20 mg was repeated. Although the symptom was relieved for a few minutes, she started to hyperventilate again after the effect of propofol wore off. This time, after an episode of hyperventilation, she developed apnea. She was unresponsive to verbal and painful stimuli. Oxygen saturation started to fall and reached up to 80%. Oxygenation and ventilation was quickly supported with bag and mask ventilation, limiting the RR to 10 breaths/min. After about 10 manual breaths, she resumed spontaneous respiration but soon started to hyperventilate. The series of hyperventilation, breath holding, desaturation, bag and mask ventilation and resumption of spontaneous respiration occurred for 3 cycles. Meanwhile, blood sample was sent for arterial blood gas (ABG) analysis. IV haloperidol 2.5 mg was given and repeated after 5 min. Finally, the patient became sedated and calm. HR settled to 90 beats/min and respiratory pattern normalized with RR of 14–16 breaths/min. Thirty minutes later, she regained consciousness. This time her respiratory pattern and vital signs continued to remain normal.

The ABG analysis revealed severe respiratory alkalosis with pH 7.672, pCO_2_ 13.6 mmHg, pO_2_ 129 mmHg, HCO_3_^−^ 18.9 mEq/L, base excess − 1.7 mmol/L and lactate 3.1 mmol/L. Electrolytes and blood glucose were within normal range. She was shifted to ICU for monitoring and further management. Haloperidol 2.5 mg IV was advised as needed for recurrence of symptom. During her stay in ICU, she remained conscious, cooperative, and had stable vital signs. Deranged ABG returned to normal. When enquired later about her experience of the event, she remembered that she felt difficulty in breathing. Apart from that she did not have any intraoperative recall or any memory of events in the PACU.

Psychiatric consultation suggested a diagnosis of acute stress reaction. No significant personal or family history of conversion disorder, panic attack, schizophrenia, depression or mania was found. She was advised oral lorazepam at bedtime. The next day she was shifted to inpatient unit and discharged from the hospital on the third postoperative day.

## Discussion and conclusion

Here we report a case of severe hyperventilation leading to apnea and desaturation in the recovery room. The exact reason for the hyperventilation was not identified despite ruling out its possible causes and treating the probable organic causes such as pain, full bladder, inadequate reversal of neuromuscular blockade, hypoxia, hypoglycemia or metabolic acidosis. However, we felt that conversion disorder was the most likely cause for this hyperventilation.

Hyperventilation in the postoperative period might be due to an effect on the respiratory center of the brain or due to a metabolic cause like metabolic acidosis. One such case reported the effect of normal saline used as irrigation solution during neurosurgery which altered the brain pH leading to hyperventilation [1]. In our case there was no reason for such metabolic derangement. Although normal saline was used for irrigation of the mastoid cavity, there was no exposure to the central nervous system. Moreover, capnography was monitored throughout the intraoperative period and ETCO_2_ was maintained in the normal range. The patient was ASA PS I, not on any medication; therefore, other systemic or pharmacological causes of metabolic derangement was less likely. The ABG analysis also ruled out the possibility of metabolic acidosis as a primary acid-base disorder. Also, hyperventilation was present only when the effect of sedation started to wear off and it reverted to normal breathing once the patient was sedated; thus neurological cause of hyperventilation was unlikely.

Emergence agitation can occur in the PACU. It is more common in children with nearly 30% of them presenting with restlessness, agitation and disorientation. It usually occurs within 10 min of recovery and resolves quickly, followed by an uneventful course. Emergence agitation is a possibility in this case as she is a teenager and the onset was approximately 15 min following recovery from anesthesia. She was anxious preoperatively, and anesthesia was maintained with isoflurane, both of which are known risk factors for emergence agitation [2]. However, our patient presented predominantly with severe hyperventilation as the primary symptom, which has not been reported as a symptom of emergence reaction in the literature. The hyperventilation was severe enough to cause severe respiratory alkalosis, apnea and desaturation, which is a rare feature in emergence agitation.

Our patient responded to haloperidol, and therefore, the possibility of postoperative delirium (POD) cannot be ignored. However, in our case there were no identified perioperative triggers that might have caused POD [2]. Moreover, postoperative delirium is more common on 1st or 2nd postoperative day and it persists for a longer period. On the contrary, our patient had an event immediately following the surgery and it lasted for a brief period of time. Also, once she recovered from the initial insult she did not have any fluctuation in the mental status or attention deficits. Thus it is less likely to be POD.

After ruling out all the possible causes, we suspected it to be conversion disorder, since it is more common in young females. There are several case reports of conversion disorder presenting during the postoperative period with symptoms like paraplegia, headache, visual loss, non-epileptic seizures and hyperventilation [3–7]. But these patients had risk factors and occurrence of such events in the past. Our patient did not give any supportive history, nor did she manifest any further episode during her stay in the hospital. The psychiatrist denied the diagnosis of conversion as this was the first episode of such nature and there were many confounding factors such as use of multiple anesthetic drugs, stress of surgery, etc. The features also did not fit the diagnostic criteria for panic attack [8]. So, the psychiatrist suggested acute stress reaction to be a more likely cause.

**Fig. 1:**
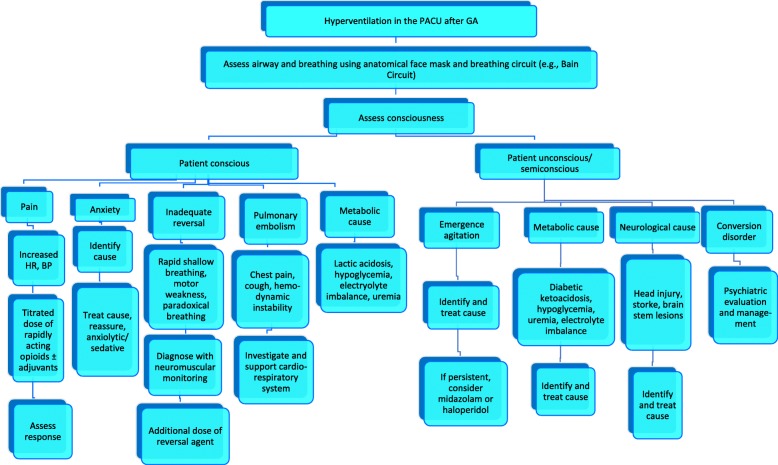
Algorithmic approach to a patient with hyperventilation in PACU after general anesthesia. Abbreviations: BP: blood pressure, GA: general anesthesia, HR: heart rate, PACU: post-anesthesia care unit

Although we could not identify the exact reason for hyperventilation, we were able to manage it successfully without any further complication. In any case with hyperventilation during the immediate postoperative period, a step by step approach is needed to identify its cause and manage accordingly. Therefore, here we have proposed an algorithmic approach to manage such patients in the PACU (Fig. 1).

## Data Availability

Not applicable.

## Abbreviations

ABG: Arterial blood gas analysis
ASA PS: American Society of Anesthesiologists Physical Status
BP: Blood pressure
ETCO_2_: End tidal carbon dioxide
HR: Heart rate
IV: Intravenous
PACU: Post-anesthesia care unit
RR: Respiratory rate
